# Early onset of cannabis use and violent behavior in psychosis

**DOI:** 10.1192/j.eurpsy.2020.71

**Published:** 2020-07-16

**Authors:** Valerie Moulin, Luis Alameda, David Framorando, Philipp-S Baumann, Mehdi Gholam, Jacques Gasser, Kim-Q Do Cuenod, Philippe Conus

**Affiliations:** 1 Department of Psychiatry, Unit for Research in Legal Psychiatry and Psychology, Institute of Forensic Psychiatry, Lausanne University Hospital (CHUV), Lausanne, Switzerland; 2 Department of Psychosis Studies, Institute of Psychiatry, Psychology & Neuroscience, King’s College London, London, UK; 3 Department of Psychiatry, Unit for Research in Schizophrenia, Center for Psychiatric Neuroscience, Lausanne University Hospital (CHUV), Lausanne, Switzerland; 4 Department of Psychiatry, Hospital Universitario Virgen del Rocío, Universidad de Sevilla, Sevilla, Spain; 5 Instituto de Investigacion Sanitaria de Sevilla, IBiS, Sevilla, Spain; 6 Department of Psychiatry, Service of General Psychiatry, Lausanne University Hospital (CHUV), Lausanne, Switzerland; 7 Department of Psychiatry, Center for Psychiatric Epidemiology and Psychopathology, Lausanne University Hospital (CHUV), Lausanne, Switzerland; 8 Department of Psychiatry, Unit for Research in Schizophrenia, Center for Psychiatric Neuroscience, Lausanne University Hospital (CHUV), Lausanne, Switzerland

**Keywords:** Cannabis use, psychosis, schizophrenia, violent behavior

## Abstract

**Background:**

Although evidence from psychosis patients demonstrates the adverse effects of cannabis use (CU) at a young age and that the rate of CU is high in subgroups of young violent patients with psychotic disorders, little is known about the possible effect of the age of onset of CU on later violent behaviors (VB). So, we aimed to explore the impact of age at onset of CU on the risk of displaying VB in a cohort of early psychosis patients.

**Method:**

Data were collected prospectively over a 36-month period in the context of an early psychosis cohort study. A total of 265 patients, aged 18–35 years, were included in the study. Logistic regression was performed to assess the link between age of onset of substance use and VB.

**Results:**

Among the 265 patients, 72 had displayed VB and 193 had not. While violent patients began using cannabis on average at age 15.29 (0.45), nonviolent patients had started on average at age 16.97 (0.35) (*p* = 0.004). Early-onset CU (up to age 15) was a risk factor for VB (odds ratio = 4.47, confidence interval [CI]: 1.13–20.06) when the model was adjusted for age group, other types of substance use, being a user or a nonuser and various violence risk factors and covariates. History of violence and early CU (until 15) were the two main risk factors for VB.

****Conclusions**:**

Our results suggest that early-onset CU may play a role in the emergence of VB in early psychosis.

## Introduction

Cannabis is the most widely used illicit recreational drug among young people (18–25 years) [1] and in patients with psychosis [1,2]. Recent meta-analyses have shown high rates of cannabis use (CU), ranging from 29 to 38% [3], in the early phase of psychosis, which can have deleterious consequences at both clinical and neurobiological levels. Substantial research [3–5] support the hypothesis that CU, especially if begun at an early age [6–10] (between 14 and 18 years), can increase the risk of developing a psychotic disorder [11], reduce the age of onset, and worsen outcomes [2,3]. In addition, it has been suggested that CU may affect the brain structure of psychotic patients [12], possibly through a global impact on the volume of both gray and white matter established at all stages of the disorder, ranging from ultra-high risk to first episode in schizophrenia patients [12].

Furthermore, several studies have highlighted the risk of violent behavior (VB) during the early phase of psychosis [13–16], and a large body of research has shown that substance use (encompassing various substances without distinction: cannabis, alcohol, and other drugs) is a major risk factor for VB in psychosis and early phase of psychosis [14,15,17–21]. In addition, the rate of CU is often high in subgroups of violent patients (VPs) with psychotic disorders [13,22–26]. However, research exploring the links between CU considered specifically and VB in psychosis is still rare [23, 24,27–32] and their results are controversial due to methodological limitations and because studies have not always controlled for important confounding risk factors [31,33–35].

In addition, despite clinical and neurobiological evidence demonstrating the adverse effects of CU at a young age in psychosis, the impact of age at onset of CU on VB has not been well explored either in these patients. Although we observed in a previous paper that VPs had started using cannabis at an early age [27], and although studies in nonpsychotic adolescents have demonstrated a link between early CU and violence [36], only one study by Cooper et al[22], has focused specifically on the issue of age at onset of CU in violent prisoners. Their results showed that prisoners at high risk of developing psychosis started using cannabis before the age of 15, but the authors did not specifically explore the link between violence and early onset of consumption. An impact of age at onset of CU on the later occurrence of VB makes sense considering the impact of CU during adolescence, a critical period for brain maturation [37], which may in turn affect structures involved both in impulsivity and VB [38,39]. Considering these elements, we decided to explore the impact of the age of onset of CU on the risk of displaying VB in a cohort of early psychosis patients.

## Methods

### Participants

Patients were recruited from the Treatment and Early Intervention in Psychosis Program (TIPP), a specialized early psychosis program implemented at the Department of Psychiatry CHUV in Lausanne, Switzerland [40,41] since 2004. Entry criteria to the program are (a) age 18–35 years, (b) residence in the catchment area, and (c) meeting threshold criteria for psychosis, as defined by the “psychosis threshold” subscale of the Comprehensive Assessment of At Risk Mental States Scale (CAARMS) [42]. Exclusion criteria are (a) antipsychotic medication for more than a total of 6 months, (b) psychosis related to intoxication or organic brain disease, or (c) an intelligence quotient <70. Patients were assessed at baseline and every 6 months over 36 months. The first 260 patients who fulfilled criteria and had sufficient data for analysis were included in the study.

The Research and Ethics Committee of the Faculty of Biology and Medicine of Lausanne University granted access to TIPP clinical data for research purposes over the study period.

### Measures

#### Assessment of substance use

Over the first few months of treatment, information was gathered regarding the age at onset of consumption and lifetime use of any substance, including alcohol, cannabis, other drugs (opioids, cocaine, hallucinogens, and others), and polysubstance, which were coded as occasional or regular by case managers at the moment of entry to the TIPP and at each time point of the follow-up. Current use was assessed by case managers (CMs) upon patient entry to the TIPP, based on the Case Manager Rating Scale (CMRS) [43]. The CMRS rates the intensity of substance use on a scale from 1 to 5 (1 being the absence of substance use and 5 being very severe substance use).

#### Diagnostic assessment

Diagnosis was the result of an expert consensus (between a psychiatrist and a psychologist) and based on the following elements: (a) diagnosis reported by treating psychiatrists in all medical documents and at the end of any hospitalization and (b) longitudinal assessment by clinical CMs [44]. In this study, the main diagnoses according to the The Diagnostic and Statistical Manual of Mental Disorders–Fourth Edition (DSM-IV) [45] were taken into account and were subdivided into five classes (see Table 1).

**Table 1.** Descriptive characteristics of the study sample and substances according to violent and nonviolent patients.

Descriptive characteristics	Cohort		Violent behavior Physical aggression		*p* value
	% (*n*) or *M* (SD)	*N* = 250	Violent (*N* = 62)	Nonviolent (*N* = 188)	
Gender (male)	% (*n*)	31.6 (79)	87 (54)	37.77 (71)	0
Age at the entry in the program	M(SD)	23.97 (0.31)	22.68 (0.56)	24.39 (0.36)	0.0115
Civil status: single	% (*n*)	84.43 (206)	85.25 (52)	84.15 (154)	
Civil status: married	% (*n*)	9.02 (22)	8.2 (5)	9.29 (17)	0.9450
Civil status: divorced	% (*n*)	3.69 (9)	3.28 (2)	2.73 (5)	
Number of years at school	M(SD)	9.79 (0.19)	8.79 (0.34)	10.1 (0.21)	0.0017
Student	% (*n*)	19.6 (49)	11.29 (7)	22.34 (42)	
Professional activity	% (*n*)	14 (35)	9.68 (6)	15.43 (29)	0.0501
No professional activity	% (*n*)	66.4 (166)	79.03 (49)	62.23 (117)	
Previous violence	% (*n*)	14.35 (31)	35.71 (20)	6.88 (11)	<0.0001
School maladjustment	% (*n*)	31.6 (79)	41.94 (26)	28.19 (53)	0.0627
Duration of untreated psychosis	M(SD)	1.27 (0.14)	1.04 (0.22)	1.34 (0.17)	0.2698
Diagnosis					
Schizophrenia	% (*n*)	58.8 (147)	62.9 (39)	57.45 (108)	
Schizophreniform/brief	% (*n*)	10 (25)	4.84 (3)	11.7 (22)	
Schizoaffective disorder	% (*n*)	9.2 (23)	14.52 (9)	7.45 (14)	0.3252
Major depression with psychotic features	% (*n*)	4.4 (11)	3.23 (2)	4.79 (9)	
Bipolar disorder	% (*n*)	6.8 (17)	6.45 (4)	6.91 (13)	
Others	% (*n*)	10.8 (27)	8.06 (5)	11.7 (22)	
Substances	%/(n) or *M* (SD)	*N* = 250	Violent *N* = 62	Non violent *N* = 188	*p* value
History of use					
Alcohol					
Age of onset	*M* (SD)	15.72 (0.17)	15.12 (0.26)	15.91 (0.2)	0.0172
Alcohol use	% (*n*)	82 (205)	79.03 (49)	82.98 (156)	0.6095
Cannabis					
Age of onset	*M* (SD)	16.47 (0.29)	15.29 (0.45)	16.97 (0.35)	0.004
Cannabis use	% (*n*)	64.4 (161)	79.03 (49)	59.57 (112)	0.0087
Other drugs					
Age of onset	*M* (SD)	18.43 (0.51)	17.84 (0.95)	18.72 (0.61)	0.4425
Other drugs use	% (*n*)	28.4 (71)	31.94 (26)	23.94 (45)	0.0104
Polysubstance					
Age of onset	*M* (SD)	19.71 (1.52)	20 (3)	19.6 (1.99)	0.9218
Polysubstance use	% (*n*)	4.8 (12)	8.06 (5)	3.72 (7)	0.2964
Program entry					
Alcohol					
Alcohol use	% (*n*)	70.21 (165)	72.41 (42)	69.49 (123)	0.7972
Cannabis					
Cannabis use	% (*n*)	42.92 (100)	63.79 (37)	36 (63)	<0.0001
Other Drugs*					
Other Drugs use	% (*n*)	8.3 (19)	14.29 (8)	6.36 (11)	0.1117
Polysubstance					
Polysubstance use	% (*n*)	1.2 (3)	3.23 (2)	0.53 (1)	0.3092

Abbreviation: SD, standard deviation.

* Opioids, cocaine, hallucinogens, and others.

#### Identification of episodes of violent behavior

Episodes of VB were identified in three distinct ways. First, CMs recorded information regarding the occurrence of violent offenses (Swiss Criminal Code) and VB (such as assault and battery) in a specific chapter of the baseline questionnaire, a reliable method considering the meta-analysis of Winsper et al[16], which showed good reliability and validity in the self-reporting of serious aggression. Second, CMs gathered any additional information through contact with parents, significant others and forensic psychiatric services (heteroreporting of aggression) over the entire duration of treatment. Finally, episodes of VB occurring during the treatment phase were identified on the basis of the Staff Observation Aggression Scale (SOAS-R [46]), which lists all critical events related to a VB during hospitalizations [47].

At the time of this study, 265 patients had been followed-up prospectively over 36 months, and they were dichotomized based on the presence or absence of VB. The group of VPs (*N* = 72) had committed physical aggression against people at least once, either before and/or during the program (time of occurrence), that met the definition of “*serious violence*”, that is, “*as assault causing any degree of injury, any use of a weapon or any sexual assault. The term any was used when the severity of the violence was not specified”* [14].

**Figure 1.**
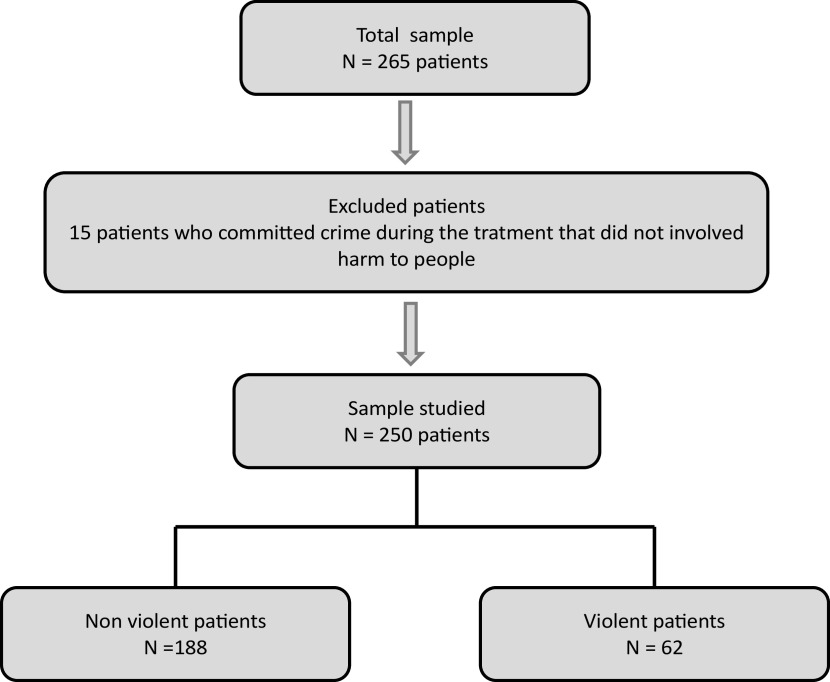
Flow chart.

To explore the impact of the age of onset of CU on the occurrence of VB during the program, we considered as VPs those who displayed VB against people during the treatment phase (*N* = 62 VPs during the program). The 10 patients who had a history of violence against people before entering the program (but who do not exhibit VB during the program) were considered nonviolent during the program. We excluded patients who had committed crimes during the program that did not involve harm to people (e.g., theft, drug trafficking). These patients cannot be considered completely nonviolent during the treatment, they were excluded from the nonviolent patient (NVP) group and were therefore not included in the analysis (*N* = 15; thus, 250 patients in total were ultimately included) (Figure 1).

#### Static risk factors and demographic risk factors of violent behavior

To account for potential confounding factors in the regression analyses, we considered the following static VB risk factors (i.e., factors related to the past that are not dynamic and subject to change) in the literature: (a) *history of violence* (assessed using a baseline questionnaire and information through parents, others and forensic psychiatric services). All previous offenses, violence, or assaults identified were considered (i.e., theft, violence against objects, persons, and other crimes); (b) *school maladjustment*; and (c) *duration of untreated psychosis* (assessed while accounting for the time between the first appearance of psychotic symptoms and the date of entry into the program). These factors were assessed at the time of entry into the program.

In addition, we considered as covariates demographic factors that are known to have an influence on VB, such as age, sex, level of education, professional activity, and marital status [14,15,48].

### Statistical analysis

Differences between groups were analyzed using Student’s *t* tests and chi-square tests of independence or Fisher’s exact test (when cells had low frequencies). In Table 1, each categorical variable was tested in each group. Variables with several categories, such as diagnostic and activity, were tested as a multi-category variable.

We used logistic regression models to assess the link between age of onset (with cutoffs at 15, 16, 17, and 18 years of age) of substance use (for alcohol, cannabis, other drugs, and polysubstance) and VB committed during the program. For each age cutoff, patients were divided into three groups: those with consumption until the cutoff age, those with consumption after the cutoff age, and those without consumption.

In the first version of these models, the two latter groups were compared with the patients with earlier consumption (prior to the cutoff age). In a second version, we adjusted the same model for static risk factors, previous violence, and demographic data as potential confounding factors. The confidence interval (CI) for estimated parameters was calculated using the profile likelihood. The level of significance of the estimated parameters is given by the *p* value calculated using the Wald statistic. Analyses were performed using “glm” function of the R environment for statistical computing, which is included in the “stats” library.

“Polysubstance use” was excluded from regression models because the number of patients (*N* = 3) was too small to be included.

## Results

### Violent behaviors during the program

Within the initial sample of 250 patients, 62 patients had committed at least one act of physical aggression during the program). Analyses were conducted on 250 patients (188 NVPs and 62 VPs). The main VB characteristics (described previously [27,49]) were physical aggression, robbery with physical aggression, and assault and battery.

### Descriptive characteristics of the study sample and substance consumption

Compared with NVPs, VPs were more likely to be men, to have a lower level of education and no professional activity; VPs were younger at the time of admission to the program and were more likely to live alone.

While 31 patients had a history of violence before entry into the TIPP, 20 committed new violent acts during the 3-year follow-up. Hence, history of violence was significantly associated with the VP group (see Table 1).

Regarding history of substance consumption, the average age at onset of alcohol consumption was 15.91 years in NVPs and 15.12 years in VPs, with a significant difference between the groups. Lifetime alcohol use was more prevalent among NVPs. The average age at onset of cannabis consumption was 16.97 years in NVPs and 15.29 years in VPs, with a significant difference between the groups, as well as for lifetime consumption: 79.03% in VPs and 59.57% in NVPs.

The average age at onset of other drugs was 18.72 years in NVPs and 17.84 years in VPs. Lifetime use of other drugs was more prevalent among VPs. The average age at onset of polysubstance use was 19.6 years in NVPs and 20 years in VPs.

Regarding the rate of substance use at program entry, there was a significant difference in CU between the groups; there were no significant differences between the groups for alcohol, other drugs, and polysubstance. These results are shown in Table 1.

### Relationship between age of onset of substance use and violent behavior during the program

**Figure 2.**
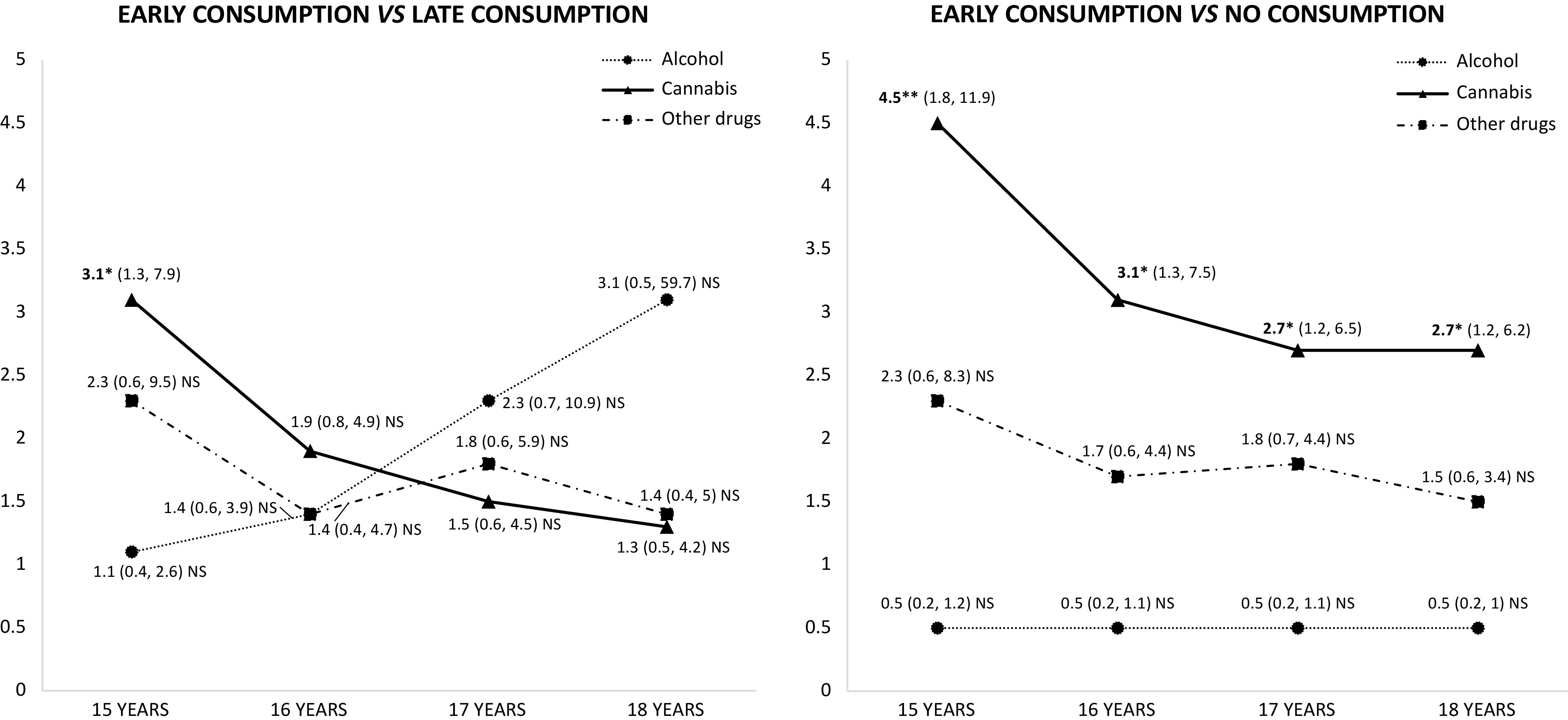
Odd ratio and age of onset of substance use. Significant results in bold, ^*^p < 0.05; ^**^p < 0.01.

The logistic regression model between the age of onset of substance use (until the cutoff age, after the cutoff age, and no consumption) and VB during the program for each substance (alcohol, cannabis, and other drugs) supported a statistically significant association: on the one hand, VB was related to CU until age 15 versus after age 15 (odds ratio [OR] = 3.1; *p* < 0.01), and on the other hand, VB was related to CU until ages 15, 16, 17, and 18 versus no consumption (Figure 2).

Adjustment of the previous logistic regression models for static factors, history of violence and possible confounders showed that only CU until age 15 (vs. nonusers) remained significantly linked to VB (OR = 4.47, *p* < 0.03). Lack of professional activity for age 16 and 18 were risk factors. For all age, a history of violence was associated with VB, with ORs ranging from 5.83 to 6.17.( see Table 2).

**Table 2.** Relationship between various age of onset of substances use, static factors, violent behavior, and demographic data during the program.

Factors		15 years			16 years			17 years			18 years		
		Odds ratios	95 CI	*p*	Odds ratios	95 CI	*p*	Odds ratios	95 CI	*p*	Odds ratios	95 CI	*p*
(Intercept)		3.46	0.06–269.75	0.563	3.61	0.08–213.57	0.523	4.11	0.09–243.12	0.483	3.10	0.07–168.67	0.566
Male (vs. female)		2.68	0.90–9.54	0.097	2.65	0.89–9.40	0.099	2.66	0.89–9.46	0.099	2.66	0.88–9.56	0.101
Age (Years)		0.91	0.81–1.02	0.117	0.92	0.82–1.02	0.139	0.92	0.82–1.02	0.134	0.92	0.82–1.03	0.175
Level of school		0.88	0.73–1.04	0.144	0.87	0.72–1.03	0.100	0.86	0.72–1.02	0.093	0.86	0.72–1.02	0.085
School maladjustement		1.70	0.68–4.28	0.254	1.65	0.66–4.13	0.279	1.65	0.65–4.16	0.284	1.72	0.69–4.32	0.241
No professional activity (vs. professional activity and student)		2.73	0.99–8.47	0.063	**2.86**	1.05–8.79	**0.049**	2.80	1.04–8.53	0.052	**2.92**	1.08–8.92	**0.043**
Marital status: single (vs. married and divorced)		0.33	0.08–1.28	0.106	0.36	0.09–1.33	0.120	0.37	0.10–1.39	0.139	0.40	0.11–1.51	0.173
Duration of untreated psychosis		0.95	0.76–1.15	0.604	0.95	0.76–1.16	0.603	0.94	0.75–1.14	0.534	0.93	0.75–1.13	0.503
History of violence		**6.17**	2.12–19.27	**0.001**	**6.23**	2.18–19.01	**0.001**	**5.98**	2.07–18.53	**0.001**	**5.83**	2.01–18.03	**0.001**
Alcohol	Early versus late consumption	0.77	0.20–2.83	0.703	1.16	0.32–4.41	0.818	1.88	0.30–16.91	0.528	3.08	0.31–77.53	0.393
	Early versus no consumption	0.27	0.06–1.07	0.071	0.31	0.08–1.10	0.076	0.31	0.08–1.04	0.065	0.30	0.08–1.01	0.057
Cannabis	Early versus late consumption	2.22	0.61–8.78	0.236	1.44	0.43–4.96	0.552	0.88	0.20–4.02	0.860	0.75	0.14–3.99	0.733
	Early versus no consumption	**4.47**	1.13–20.06	**0.039**	3.28	0.94–13.05	0.073	2.91	0.86–11.14	0.098	2.95	0.88–11.19	0.092

Significant results in bold, *p* < 0.05.

a Opioids, cocaine, hallucinogens, and others.

## Discussion

To our knowledge, this is the first study to explore the links between the precocity of multiple substance use, including CU, and subsequent VB in early phase of psychosis. Besides confirming previous literature [14,19,20,48], regarding history of violence and lack of professional activity as risk factors for VB during treatment, our main findings are as follows: first, patients with VB started using cannabis at a significantly earlier age than patients without such behaviors. Second, early-onset CU was a risk factor for VB when the model was adjusted for each age group, other types of substance use, being a user or a nonuser and various static VB risk factors. Third, history of violence and CU until age 15 were the two main risk factors for VB.

In our EPP cohort, 64% of the patients had a history of lifetime CU, which is consistent with other studies (range 24–74%) [3,50–52]. This percentage was significantly higher (79%) for patients in the VP group. In line with Cooper et al[22], the mean age at which patients in the VP group began using cannabis was 15 years while it was 17 years in the NVP group (a figure consistent with previous studies [8,53–55]). At program entry, while violent patients are 22 years old on average, the rates of CU remained significantly higher in VPs than in NVPs. These results highlight the specific characteristics of the VP group regarding the precocity, prevalence, and persistence of CU.

Research has shown a link between CU before 15 years of age and the psychopathological course of psychotic patients [6,8,9] and that this link was specific to cannabis, as opposed to other substances [6]. Our results suggest a similar pattern regarding VB. Indeed, among all patients who had used substances, the likelihood of manifesting VB was four times higher when CU started before age 15 (after adjustment for static factors). Consistent with a recent study [29], our results suggested that alcohol use was not associated with VB. These findings could be due to the lower lifetime alcohol consumption prevalence we observed in VPs compared to NVPs. However, the effects of these substances on VB require further investigation and replication in other cohorts.

In line with previous literature [14,20,48], our results have shown that history of violence was a major risk factor for VB during the program, and that CU prior to age 15 was the second most important factor. Our results are consistent with studies that have exposed the impact of social environment on juvenile delinquency, and the effects of early CU and delinquency Institute of Forensic Psychiatry [34,36]. Studies suggest that the association of deviant peers and delinquents may promote early substance use [56]; other research, suggests that early CU may lead to delinquency and illicit trafficking [57]. These findings are in line with research showing a distinctive profile of VP in psychosis, with a complex developmental trajectory characterized by early conduct disorders (prior to age 15), associated with substance use and antisocial behavior [58–61]. Early CU would be an integral part of these young’s lives and would be a key variable linking delinquency in the early and late stages of life. Patients in this trajectory would exhibit violent behavior prior to and after the onset of psychotic disorder. VB after the onset of psychosis would be related to the combination of anti-social conduct and substance use present from adolescence onward (independent of psychotic disorders).

## Limitations and Strengths

First, substance use prior to entry into the program is based on information provided by patients and it may be imprecise. Second, patients may have been using various doses of cannabis, different types of cannabis with varying proportions of THC, or cannabidiol, known for their modulating impact in psychosis [2]. Third, although we used different methods to assess the occurrence of VB (self-report by patients, heteroreport by relatives, information stemming from forensic services and a standardized assessment tool), the occurrence of VB prior to the program might have been under-evaluated due to a lack of access to police and criminal records. Fourth, the sample was relatively small and these results need replication in larger cohorts.

## Conclusions

Our results suggest that early-onset CU may play a role in the emergence of VB in early phase of psychosis. Moreover, information campaigns [62] aimed at young adolescents and the general population should raise awareness of the possible effects of early CU. Finally, age at onset of CU might inform clinicians regarding the need to propose specific treatment in order to prevent VB in such patients.

## Data Availability

The data that support the findings of this study are not openly available.
